# Supplementary material for: CycloPepper: a machine learning platform for predicting cyclization outcomes and optimizing synthesis of therapeutic cyclopeptides
Source: Nat Commun. 2026 Feb 14;17:2803. doi: 10.1038/s41467-026-69441-w (PMC13021993; doi:10.1038/s41467-026-69441-w)

Supplementary Information

for

CycloPepper: A Machine Learning Platform for Predicting Cyclization Outcomes and Optimizing Synthesis of Therapeutic Cyclopeptides

Yourong Pan,[1,2,†] Chengrui Hu,[1,2,†] Jiaqi Li,[3] Feng Wan,[1,2] Xin Hong[3,4] and Chengxi Li[1,2*]*

[1] Key Laboratory of Biomass Chemical Engineering of Ministry of Education, College of Chemical and Biological Engineering, Zhejiang University, Hangzhou, 310027, P.R. China.

[2] Zhejiang Key Laboratory of Intelligent Manufacturing for Functional Chemicals, ZJU-Hangzhou Global Scientific and Technological Innovation Center, Zhejiang University, Hangzhou, 311215, P.R. China.

[3] Center of Chemistry for Frontier Technologies, Department of Chemistry, Zhejiang University, Hangzhou, 310027, P.R. China

[4] School of Chemistry and Chemical Engineering, Henan Normal University, Xinxiang 453007, P.R. China

[†]These authors contributed equally to this work.

*Corresponding authors (email: hxchem@zju.edu.cn and cxli@zju.edu.cn)

Table of Contents

1. General information--3

1.1 Reagents and solvents --3

1.2 Analytical protocols--3

2. Preparation of cyclic peptides via an automated flow-based instrument ---4

2.1 Introduction of automated flow synthesizer 'CycloBot' -----------4

2.2 Introduction of control software 'AutoXpert' ----------------------6

2.3 Synthesis of DAN resin ---7

2.4 Automated cyclic peptide synthesis ---------------------------------7

2.5 Details of cyclic peptide dataset construction --------------------- 10

3. Feature engineering and model selection --------------- 16

3.1 Details of peptide sequences encoding ---------------------------- 16

3.2 Details of machine learning model -------------------------------- 17

3.3 Details of model training-- 19

4. Details of the validation experiment -------------------- 20

4.1 Sequences of validation experiment---------------------------------- 20

4.2 Details of sequence similarity evaluation---------------------------- 21

5. Introduction for the functions of "CycloPepper"------ 24

6. Procedures for predicting cyclization sites of cyclic peptides targeting various diseases ----------------------------- 26

7. References -- 35

8. Synthetic LC-MS data for validation experiments ---- 37

1. General information

1.1 Reagents and solvents

Unless otherwise noted, commercial reagents were used without further purification. *N,N*-Dimethylformamide (DMF, 99.8%, water content < 50 ppm, determined by Karl Fischer titration) was purchased from Macklin Industrial Corporation. Dichloromethane (DCM) and acetonitrile (MeCN, HPLC grade) were acquired from ASTOON Chemical Technology, Inc. (Wilmington, Delaware).

Fmoc-protected amino acids, including Fmoc-Ala-OH, Fmoc-Arg(Pbf)-OH, Fmoc-Asn(Trt)-OH, Fmoc-Asp(^{t}Bu)-OH, Fmoc-Cys(Trt)-OH, Fmoc-Gln(Trt)-OH, Fmoc-Glu(^{t}Bu)-OH, Fmoc-Gly-OH, Fmoc-His(Trt)-OH, Fmoc-Ile-OH, Fmoc-Leu-OH, Fmoc-Lys(Boc)-OH, Fmoc-Met-OH, Fmoc-Phe-OH, Fmoc-Pro-OH, Fmoc-Ser(^{t}Bu)-OH, Fmoc-Thr(^{t}Bu)-OH, Fmoc-Trp(Boc)-OH, Fmoc-Tyr(^{t}Bu)-OH, Fmoc-Val-OH were obtained from BidePharm. *O*-(7-Azabenzotriazol-1-yl)-*N,N,N',N'*-tetramethyluronium hexafluorophosphate (HATU, ≥98.0%) was also sourced from BidePharm in China. AldraAmine trapping agents (suitable for 1000–4000 mL DMF, catalog number Z511706), diisopropylethylamine (DIEA, 99.5%), piperidine (ACS reagent, ≥99.0%) were purchased from Sigma-Aldrich. Rink Amide-AM resin (0.3-0.5 mmol/g loading, 100–200 mesh) was obtained from Sunresin New Materials Co., Ltd., Xi'an, China. Isoamyl nitrite (95%) was supplied by Energy Chemical Co. Ltd. Formic acid (FA, ≥95.0%) was purchased from Sigma-Aldrich.

1.2 Analytical protocols

Liquid chromatography-mass spectrometry (LC-MS) data were acquired using a Shimadzu LCMS-2050 system. The filtered reaction solution was directly used for following LC-MS testing. The mobile phases utilized for LC-MS consisted of 0.1% formic acid in water (solvent A) and acetonitrile (solvent B). MS conditions: positive electrospray ionization (ESI) in extended dynamic mode, with a mass range of 100–2000 m/z.

Method A (8 min): LC conditions: Shimadzu Shim-pack GIST C18 column (2 μm, 2.1 × 100 mm), temperature: 40 °C, gradient: 0-0.5 min, 5-65% B; 0.5-5.0 min, 65-90% B; 5.0-7.4 min, 90% B; 7.4-8.0 min, 5% B. Flow rate: 0.3 mL/min. The total runtime was 8 minutes. MS detection was conducted from 0 to 8 minutes.

Method B (10 min): LC conditions: Shimadzu Shim-pack GIST C18 column (2 μm, 2.1 × 100 mm), temperature: 40 °C, gradient: 0-0.5 min, 5%-60% B; 0.5-9 min, 60-80% B; 9-10.0 min, 5% B. Flow rate: 0.3 mL/min. The total runtime was 10 minutes. MS detection was conducted from 0 to 10 minutes.

2. Preparation of cyclic peptides via an automated flow-based instrument

2.1 Introduction of automated flow synthesizer 'CycloBot'

CycloBot[1] is designed and produced for automated cyclic peptide synthesis, which can be roughly divided into seven modules: central control module, reagents/solvents module, delivery module, heating module, reaction module, online UV module, and collection module **(Supplementary Fig. 1)**.

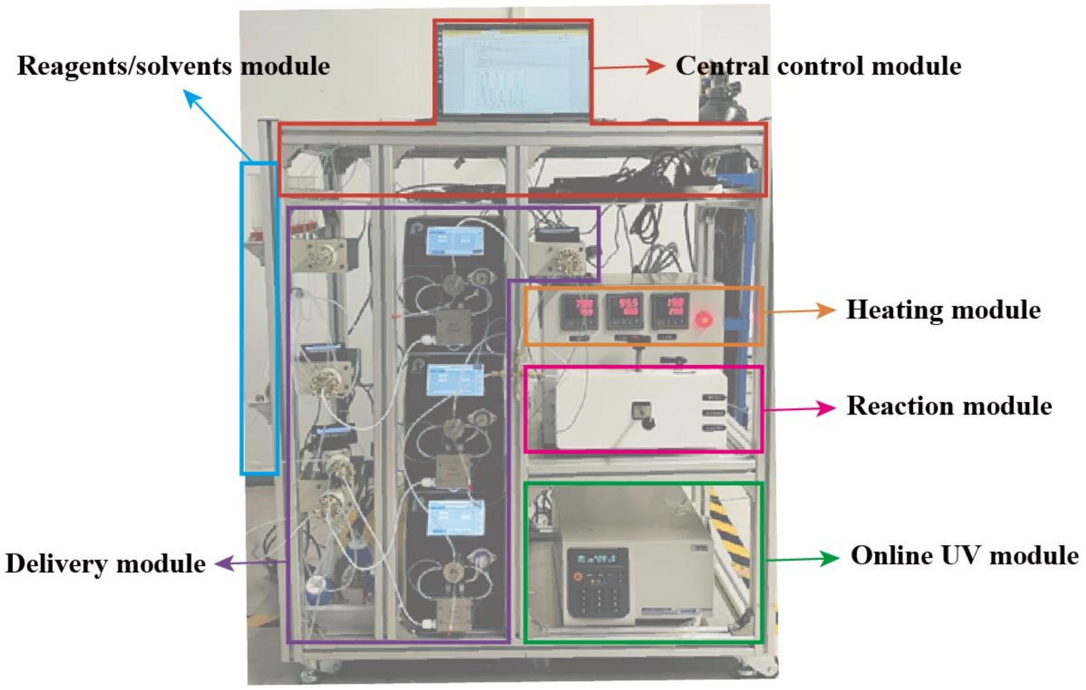

Supplementary Fig. 1. Overview of modules of the CycloBot automated synthesis system.

Central control module

The central control module is controlled by a laptop with AutoXpert software, which manipulates all VICI valves, HPLC pumps, and UV monitors, and also receives signals from UV monitors.

Reagents/solvents module

Deprotection solution, amino acid solution, HATU solution, coupling base solution, activated reagent, cyclization reagent, and DMF are stored in Schott brand media bottles or injection syringes.

Delivery module

The delivery module consists of three HPLC pumps for the solution and DMF delivery, three 24-position VICI valves for reagents, and DMF switchover.

Heating module

The heating module is part of the reactor, which offers three channels at different temperatures **(Supplementary Fig. 2)**. In the synthesis process, three channels offer different temperatures for different uses: Channel A is 90 °C, channel B is at room temperature (25 °C), and channel C is at 50 °C.

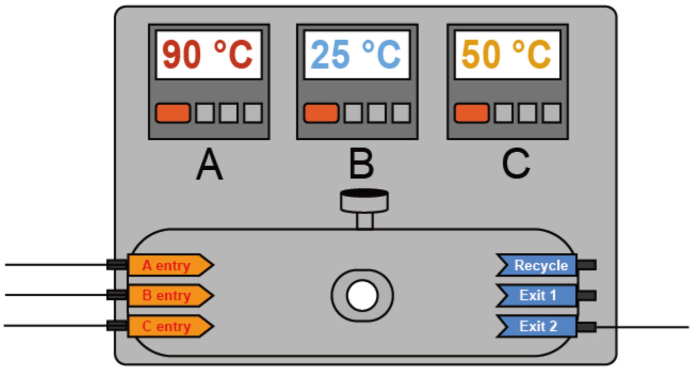

Supplementary Fig. 2. Overview of the heating module.

Reaction module

The reaction module is part of the reactor. The resin is placed in the microreactor which is suitable for synthesis of 20-50 mg scale of resin.

Online UV module

The wavelength of the UV detector ranges from 200 to 400 nm. The effluent is monitored at 310 nm and 220/205 nm under programmed time control.

Collection module

The collection module is divided into a product collection bottle and a waste collection bottle.

2.2 Introduction of control software 'AutoXpert'

AutoXpert[1], a graphical interface control software, was designed to manage the workflow and monitor synthesis efficiency online. It was developed to provide experimenters without programming backgrounds the flexibility to control the synthesis platform.

Control of the synthesizer encompasses apparatus configuration, protocol construction, protocol execution, and real-time monitoring. These functions are organized into three separate modules, including apparatus configuration, protocol configuration, and experiment execution. Each module has its window, accessed through a unified interface **(Supplementary Fig. 3)**. The apparatus configuration module is designed for configuring devices. The core configuration information includes the serial port number for each active component, which is used by the computer to send control commands, and the port configuration for the VICI valve. The protocol module is the core component where users create reusable synthesis schemes. The parameters of this operation unit include the orientation of four valves (three reagents and one reactor), pump flow rate, and duration. Users can assemble multiple operations and arrange them into a complete protocol. The experiment execution module is the module to execute protocols. Users add the protocol file to the execution array, and protocols in it can be executed one by one automatically.

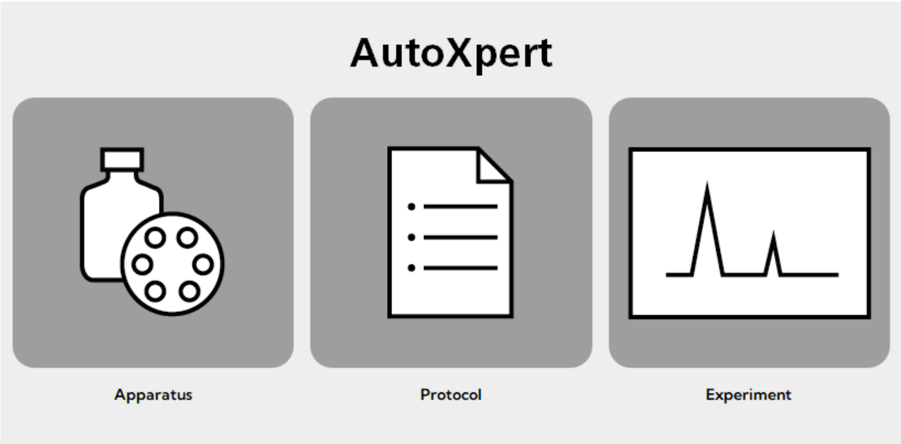

Supplementary Fig. 3. Entry interface of AutoXpert.

2.3 Synthesis of DAN resin

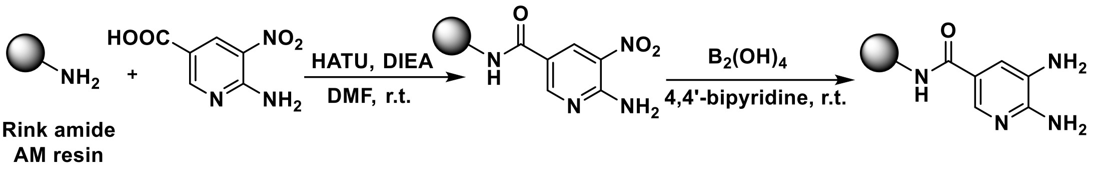

Rink Amide-AM resin (300 mg, 0.3-0.5 mmol/g loading) was placed in a 10-mL disposable reaction vessel (Torvig) equipped with a sieve plate and swollen in 5 mL of DMF. The resin was then treated with 20% piperidine (2 mL, 5 min × 2) for Fmoc deprotection, washed with DMF, and coupled with 6-amino-5-nitronicotinic acid (4 equiv) using HATU (3.8 equiv) and DIEA (8 equiv) in 2 mL of DMF for 1 hour at room temperature. Following the coupling, the resin was washed sequentially with DMF (5 mL × 3) and DCM (5 mL × 3), then dried under vacuum. Then tetrahydroxy diboron (3 equiv) and 4,4'-bipyridine (0.05 equiv) were dissolved in 2.5 mL of DMF. The solution was added to the resin above in a 10 mL disposable reaction vessel (Torvig) with a sieve plate, and shaken for 30 min until the blue solution turned yellow. After completion of the reaction, the solution was removed, and the DAN resin was washed with DCM (5 mL × 3) and DMF (5 mL × 3), then dried under vacuum[2]. In this project, the loading of DAN resin was calibrated as 0.36 mmol/g.

2.4 Automated cyclic peptide synthesis

The synthesis process includes three stages: chain growth, activation, and cyclization.

For chain growth, after a 45 s DMF wash, reagents are delivered via pumps from the storage module, where the selected amino acid is mixed with DIEA (10% v/v) and the coupling agent HATU. The resulting mixture is directed through thermally regulated Channel A (90 °C) to facilitate active ester formation. The activated intermediate then enters the coupling module under controlled DMF flow (40 s), followed by Fmoc deprotection (36 s) after an additional 45 s DMF wash. This cycle repeats iteratively until peptide chain assembly is complete.

Following chain growth, the reactor transitions to channel B at ambient temperature (25 °C). After a 45-second wash with DMF to facilitate temperature equilibration, isoamyl nitrite is introduced to activate the DAN linker within 30 seconds.

In the final cyclization step, channel C delivers a 1% (v/v) DIEA solution at 50 °C for 180 seconds. Once cyclization is complete, the collection module is automatically switched to collect the target cyclic peptide product (**Supplementary Fig. 4**).

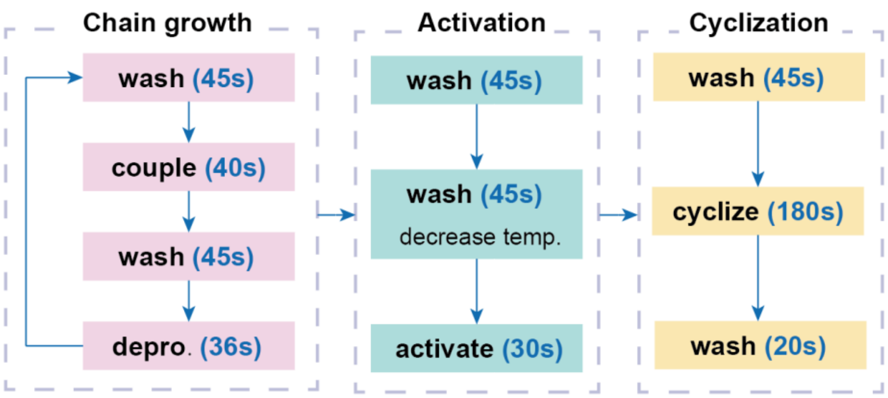

Supplementary Fig. 4. Flowchart of cyclic peptide synthesis on CycloBot.

Detailed workflow is listed in **Supplementary Table 1**. **Supplementary Fig. 5** is an overview of the flow path of CycloBot. The connection of reagents to pumps and details of solution are as follows:

Chain growing steps: Pump 1: concentration of amino acid monomer in DMF: 0.4 M, Pump 2: concentration of HATU in DMF: 0.38 M, Pump 3: DIEA: 10% in DMF (v/v), Deprotection: Pump 2: 40% piperidine and 1% HCOOH (v/v) in DMF (which would be diluted into 20%/0.5% when applied in synthesis); **Activation step**: Pump 3: 20% isoamyl nitrite in DMF (v/v), **Cyclization step**: Pump 3: DIEA: 1% in DMF (v/v), Pump 1, 2, 3: DMF for washing; Channel A: 90 °C, Channel B: 25 °C, Channel C: 50 °C. Volume refers to a single pump, if three pumps are delivered together, the total volume of solvent or solution triples.

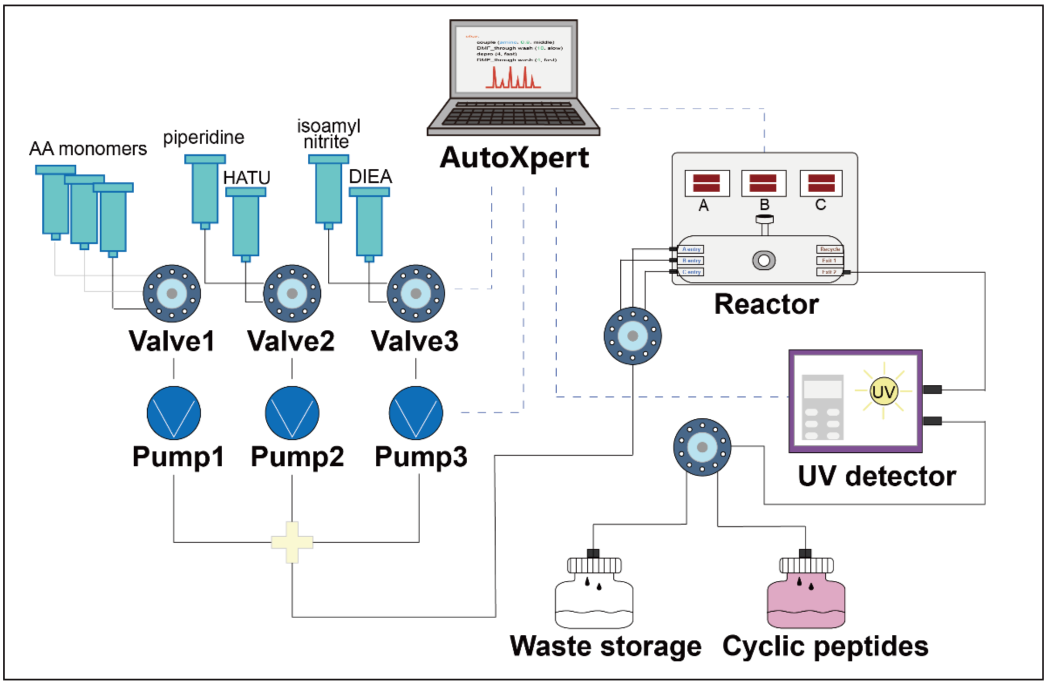

Supplementary Fig. 5. Flow path of cyclic peptide synthesis on *CycloBot*.

Supplementary Table 1 Detailed workflow of automated synthesis of cyclic peptides.

Entry	Channel	Steps	Reagents	Volume (mL)	Flow rate (mL/min)	Time (s)
1	Channel A (90 °C)	Swelling	DMF	20	10	120
2		First AA Coupling Injection	AAs, HATU, DIEA	0.4	10	2.4
3		First AA Coupling Over-injection	AAs, HATU, DIEA	0.4	3	8
4		Slow Reaction	DMF	2	3	40
5		Wash	DMF	7.5	10	45
6		Deprotection	piperidine	3	5	36
7		Wash	DMF	7.5	10	45
8		Second and other AA Coupling and Deprotection	AAs, HATU, DIEA	0.4	10	2.4
9			DMF	2	3	40
10			DMF	7.5	10	45
11			piperidine	3	5	36
12			DMF	7.5	10	45
13	Channel B (25 °C)	Cooling	DMF	15	10	45
14		Activation	Isoamyl Nitrite	5	5	30
15	Channel C (50 °C)	Wash	DMF	7.5	10	45
16		Cyclization	DIEA (1%)	15	5	180
17		Wash	DMF	3	10	20

2.5 Details of cyclic peptide dataset construction

The dataset was established through integrated synthesis and detection processes. Synthesis utilized *CycloBot* with 40 mg DAN resin (0.36 mmol/g loading) following standard protocols (refer to **Automated Cyclic Peptide Synthesis**). Post-synthesis mixtures underwent direct LC-MS analysis without processing (1 µL injection volume).

Critical cyclization success criteria mandated detection of $[M+H]^+$ or $[M+Na]^+$ parent ions for "1" classification, while absence of both parent ions yielded "0" designation. Adducted masses (e.g., $[2M+H]^+$) and fragment ions were used as supplementary validation indicators instead of primary evidence for cyclization success.

A total of 22 seed sequences (SFEGMPN, MSFEGMP, YNSFPGM, NGTKGDY, DGAFQAY, LDPGTFI, CGDYGTK, HTLGTID, FEGMPNS, RGFACAY, WRDGGTDDA, QVEDGTHYK, RHDADGGTD, SEAVDGTAK, MKWPFGPTL, NSGGTDDAD, CMILDPGTF, KRDPGTFIL, HVIGPSYFG, HCAYRGFAA, TYRGFAACA, ICWPFGPTL) were initially generated by a programmatic approach to ensure representation of all 20 proteinogenic amino acids. From this pool, subsequences were manually selected to maximize diversity in amino acid composition while retaining compatibility with the directed variation strategies, ultimately yielding a library of 306 sequences (**Supplementary Table 2**). This design ensured that the resulting variants captured the full range of structural diversity and cyclization potential, thereby rendering the dataset comprehensive and reliable.**Supplementary Table 2** 306 cyclic peptide sequences and scores.

Entry	Sequence	Score
1	RG	1
2	HG	1
3	NG	1
4	QG	1
5	FW	1
6	NY	1
7	FC	1
8	KT	1
9	PH	1
10	LR	1
11	QW	1
12	CK	1
13	AQ	1
14	MY	1

Entry	Sequence	Score
154	DGTAK	1
155	TDDAD	1
156	DPGTF	1
157	GTFIL	1
158	RGFAA	1
159	FAACA	1
160	FGPTL	1
161	VDGTA	1
162	KEFKG	0
163	EGMPN	0
164	SFPGM	0
165	PGTFI	0
166	PSYFG	0
167	TGKDG	0

Entry	Sequence	Score
15	NI	1
16	EH	1
17	RS	1
18	HK	0
19	WY	0
20	RS	0
21	GG	0
22	KG	0
23	DG	0
24	EG	0
25	SG	0
26	TG	0
27	CG	0
28	WG	0
29	PG	0
30	AG	0
31	VG	0
32	IG	0
33	LG	0
34	MG	0
35	FG	0
36	YG	0
37	WA	0
38	SV	0
39	MI	0
40	DF	0
41	EA	0
42	YV	0
43	DP	0
44	GH	0

Entry	Sequence	Score
168	TPDLG	0
169	QACYG	0
170	WAAGG	0
171	MNSFGG	1
172	TDPGFG	1
173	TDGFFG	1
174	TDFFFG	1
175	TIDTLG	1
176	AFQAYG	1
177	FCAYRG	1
178	TDGHFG	1
179	TDDADG	1
180	TFGYRG	1
181	TKGDYG	1
182	MNSFPG	1
183	GMNSGF	1
184	FCGYRG	1
185	THYKDG	1
186	TFGKDG	1
187	TGKADG	1
188	KKGGKG	1
189	QPGQPG	1
190	TIDFLG	1
191	KKKKKG	1
192	TKDADG	1
193	FEGMPN	1
194	SFEGMP	1
195	FGIGPS	1
196	GDYGTK	1
197	TLGTID	1

Entry	Sequence	Score
45	FF	0
46	AYG	1
47	TDG	1
48	TKG	1
49	FRG	1
50	GKG	1
51	GYG	1
52	SYG	1
53	TYG	1
54	QWI	1
55	GPM	1
56	QPF	1
57	STR	1
58	YQG	0
59	HCG	0
60	VMG	0
61	ILG	0
62	QYG	0
63	DHG	0
64	WNG	0
65	ERG	0
66	CLG	0
67	YHG	0
68	FTG	0
69	YQG	0
70	RVG	0
71	MAG	0
72	LDG	0
73	NFG	0
74	HRG	0

Entry	Sequence	Score
198	EGMPNS	1
199	GFACAY	1
200	GGTDDA	1
201	DGTHYK	1
202	ADGGTD	1
203	VDGTAK	1
204	GTDDAD	1
205	LDPGTF	1
206	GPSYFG	1
207	YRGFAA	1
208	GFAACA	1
209	TRKADG	0
210	PTLDFG	0
211	AMRPFW	0
212	NSFPGM	0
213	GTKGDY	0
214	GAFQAY	0
215	DPGTFI	0
216	PFGPTL	0
217	PGTFIL	0
218	PFGPTL	0
219	MPNSFEG	1
220	TDDADGG	1
221	TAKAVDG	1
222	FACAYRG	1
223	ITPGIPG	1
224	GPTLWPF	1
225	GITPVIF	1
226	GIGPSYF	1
227	MSFEGMP	1

Entry	Sequence	Score
75	VWG	0
76	PSG	0
77	AIG	0
78	EHG	0
79	WIC	0
80	FGQ	0
81	LPV	0
82	YTC	0
83	RSM	0
84	HKV	0
85	NML	0
86	KET	0
87	VFA	0
88	HCY	0
89	YYL	0
90	DCL	0
91	ESN	0
92	VQI	0
93	IQM	0
94	HYR	0
95	NKF	0
96	WYL	0
97	RVE	0
98	GTF	1
99	TDGG	1
100	TGKG	1
101	SEPG	1
102	AYKG	1
103	FGYG	1
104	FKFG	1

Entry	Sequence	Score
228	QAMRPFW	1
229	YFGIGPS	1
230	YNSFPGM	1
231	NGTKGDY	1
232	CGDYGTK	1
233	HTLGTID	1
234	FEGMPNS	1
235	RGFACAY	1
236	DGGTDDA	1
237	EDGTHYK	1
238	DADGGTD	1
239	AVDGTAK	1
240	GGTDDAD	1
241	ILDPGTF	1
242	DPGTFIL	1
243	IGPSYFG	1
244	AYRGFAA	1
245	RGFAACA	1
246	WPFGPTL	1
247	TFILDPG	0
248	SFEGMPN	0
249	DGAFQAY	0
250	LDPGTFI	0
251	GITPVIP	0
252	GITPVIG	0
253	ITPVIPG	0
254	SEPMNFG	0
255	AFQYRCG	0
256	FAQYCRG	0
257	MGPNSFEG	1

Entry	Sequence	Score
105	FGRG	1
106	FGKG	1
107	YAKG	1
108	GMPN	1
109	YGTK	1
110	GTID	1
111	MPNS	1
112	ACAY	1
113	GGTD	1
114	GTAK	1
115	SYFG	1
116	GFAA	1
117	AACA	1
118	GPTL	1
119	EGMP	0
120	RPFW	0
121	IGPS	0
122	FPGM	0
123	KGDY	0
124	FQAY	0
125	GTFI	0
126	TDDA	0
127	THYK	0
128	DDAD	0
129	PGTF	0
130	TFIL	0
131	FCGG	0
132	AYRG	0
133	TDPG	0
134	SEGG	0

Entry	Sequence	Score
258	FAACAYRG	1
259	GLPPPIFG	1
260	TDDADAGG	1
261	RDGGTDDA	1
262	HDADGGTD	1
263	EAVDGTAK	1
264	KWPFGPTL	1
265	SGGTDDAD	1
266	MILDPGTF	1
267	RDPGTFIL	1
268	VIGPSYFG	1
269	YRGFAACA	1
270	TDDADAAG	0
271	FAQYKCRG	0
272	FAQYACRG	0
273	CAYRGFAA	0
274	CWPFGPTL	0
275	TDDADAGGG	1
276	TDDADAAAG	1
277	WRDGGTDDA	1
278	RHDADGGTD	1
279	SEAVDGTAK	1
280	MKWPFGPTL	1
281	NSGGTDDAD	1
282	HVIGPSYFG	1
283	TYRGFAACA	1
284	QVEDGTHYK	0
285	CMILDPGTF	0
286	KRDPGTFIL	0
287	HCAYRGFAA	0

Entry	Sequence	Score
135	MSPG	0
136	QACG	0
137	TDDAG	1
138	MNSFG	1
139	SEMNG	1
140	FCGYG	1
141	FAYRG	1
142	KKKKG	1
143	FEGMP	1
144	MRPFW	1
145	GIGPS	1
146	TKGDY	1
147	AFQAY	1
148	DYGTK	1
149	LGTID	1
150	GMPNS	1
151	FACAY	1
152	GTDDA	1
153	GTHYK	1

Entry	Sequence	Score
288	TDDADAGFGG	1
289	TDADGGKGGG	1
290	MGGGPNSFEG	1
291	AAAAAFAYRG	0
292	TGDADGGKGGG	1
293	TDDADAAAGGG	1
294	MGGGGMPNSFEG	1
295	MGGGGGPNSFEG	1
296	PFAGPFAGPFAG	1
297	FAAAAAQYACAG	0
298	TDDTFGGKGTDG	0
299	TIDFLGTIDFLG	0
300	AAAAAAFQYRCG	0
301	MGGGGGMPNSFEG	1
302	TDDADAAAGGGGG	1
303	TDAGTDAGTDAG	0
304	DPGTFILDGTDLG	0
305	MGGGGGGMPNSFEG	1
306	TDGDTFGGKGTDGG	0

3. Feature engineering and model selection

3.1 Details of peptide sequences encoding

The original input features were 716-dimensional vectors, concatenated by 280-dimensional one-hot encoded sequences, 20-dimensional amino acid composition (AAC), 400-dimensional dipeptide composition (DPC), and 16-dimensional hand-crafted features.

Hand-crafted features were set by summarizing laboratory experience. Below are the rules summarized. When synthesizing peptides on resin, if the last amino acid is Proline (P) or Cysteine (C), most peptides fail to undergo cyclization. When the first amino acid is Glycine (G), the likelihood of obtaining cyclic peptide products is higher.

Additionally, when the first amino acid is Threonine (Thr), Methionine (Met), or Phenylalanine (Phe), and the last amino acid is G, the likelihood of obtaining a cyclic peptide product is increased. Meanwhile, inserting amino acids such as G or Alanine (A) in sequences that are capable of cyclization significantly increases the probability of obtaining cyclic peptides, such as sequence-MPNSFEG, sequence-FRG, and sequence-TDG. These sequence characteristics were then converted into one-hot encoding, forming a 16-dimensional feature vector.

The setting of hand-crafted features, along with other features, participated in the subsequent feature selection process, and ultimately only a small portion was retained. This reflects the limitations of relying solely on the experimenter's experience. However, some of the retained features also confirmed the reliability of the experimental insights.

3.2 Details of machine learning model

Model selection is a critical step in the training of machine learning models. To assess the performance of the candidate algorithms, 5-fold cross-validation was employed. Model performance was evaluated using accuracy, calculated via the accuracy_score function from the sklearn.metrics module. The candidate algorithms include 7 commonly used models from the scikit-learn package: Gaussian Naive Bayes (GaussianNB), k-Nearest Neighbors (KNN), Gradient Boosting (GB), Random Forest (RF), Support Vector Classifier (SVC), Logistic Regression (LR), and Adaptive Boosting (AdaBoost). Extreme Gradient Boosting (XGB) was imported from the xgboost package. The models were trained using default hyperparameter settings with certain modifications, as detailed in **Supplementary Table 3**. The corresponding 5-fold cross-validation performance metrics are presented in **Supplementary Table 4**.

Supplementary Table 3 Details of machine learning models.

Model name	Sklearn model names	Modified hyperparameters
**GaussianNB**	GaussianNB	--
**KNN**	KNeighborsClassifier	n_neighbors=5, p=1, weights=‘distance’
**GB**	GradientBoostingClassifier	n_estimators=100, learning_rate=0.5, max_depth=7

**RF**	RandomForestClassifier	--
**SVC**	SVC	--
**LR**	LogisticRegression	--
**XGB**	--	colsample_bytree=1.0, learning_rate=0.1, max_depth=3, n_estimators=100, subsample=1.0
**AdaBoost**	AdaBoostClassifier	n_estimators=200
**Voting1**	VotingClassifier	Voting='soft'
**Voting2**	VotingClassifier	Voting='soft'
**Voting3**	VotingClassifier	Voting='soft'
**Stacking**	StackingClassifier	--

Supplementary Table 4 Details of the classification performances.

Model name	Accuracy	Standard Deviation
**GaussianNB**	0.739	0.048
**KNN**	0.834	0.058
**GB**	0.817	0.039
**RF**	0.794	0.063
**SVC**	0.837	0.065
**LR**	0.814	0.056
**XGB**	0.758	0.057
**AdaBoost**	0.820	0.051
**Voting1**	0.788	0.067
**Voting2**	0.824	0.064
**Voting3**	0.756	0.052
**Stacking**	0.824	0.055

The differences among the learning algorithms were not statistically significant, as all performances fell within one standard deviation. The best model is SVC, which achieved the highest mean accuracy (0.84 ± 0.06).

3.3 Details of model training

The original 716-dimensional vector undergoes zero-dimensional elimination, resulting in a final dimension of 414. This 414-dimensional vector was then utilized as input for twelve different machine learning models.

To identify features significantly impacting model performance from the 414-dimensional vector, we conducted forward feature selection using 5-fold cross-validation within the training data. This process commenced with an empty feature set and iteratively added the most impactful feature based on cross-validation accuracy improvement (including MinMaxScaler in each fold). If no improvement in accuracy was observed for five consecutive rounds, the selection process was terminated. Comparing the 5-fold cross-validation results of 12 models after feature selection, the Support Vector Classifier (SVC) displayed the highest average accuracy, achieving a score of 0.84. The feature selection process concluded when 31 features were identified. Overall, the model's accuracy was enhanced from 0.61 to 0.84 **(Supplementary Fig. 6)**.

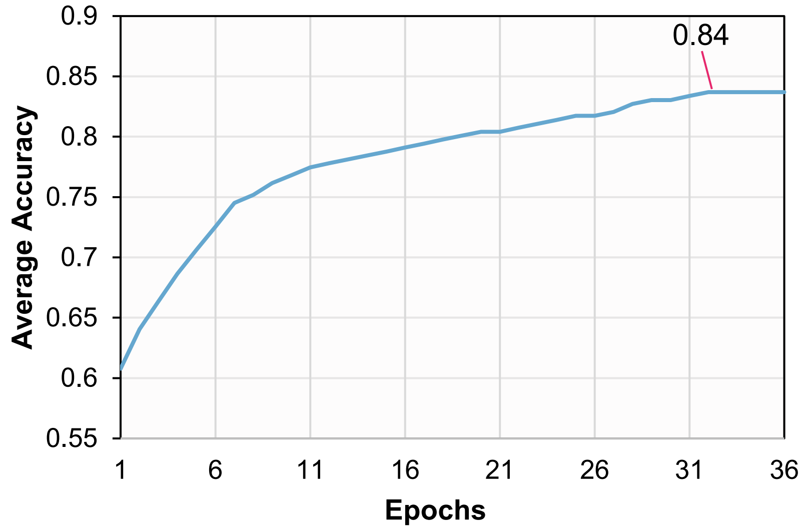

Supplementary Fig. 6. Average accuracy (n=5 folds) during epochs in forward feature selection for SVC. Source data are provided as a Source Data file.

To further evaluate the contribution of the selected features, an ablation study was performed. In this procedure, each feature was removed individually from the final feature set, and the models were retrained and evaluated using 5-fold cross-validation. Model performance was again measured by accuracy. The results showed that the

removal of any single feature led to a decrease in cross-validation accuracy, indicating that all selected features contributed positively to the predictive performance of the models **(Supplementary Fig. 7)**.

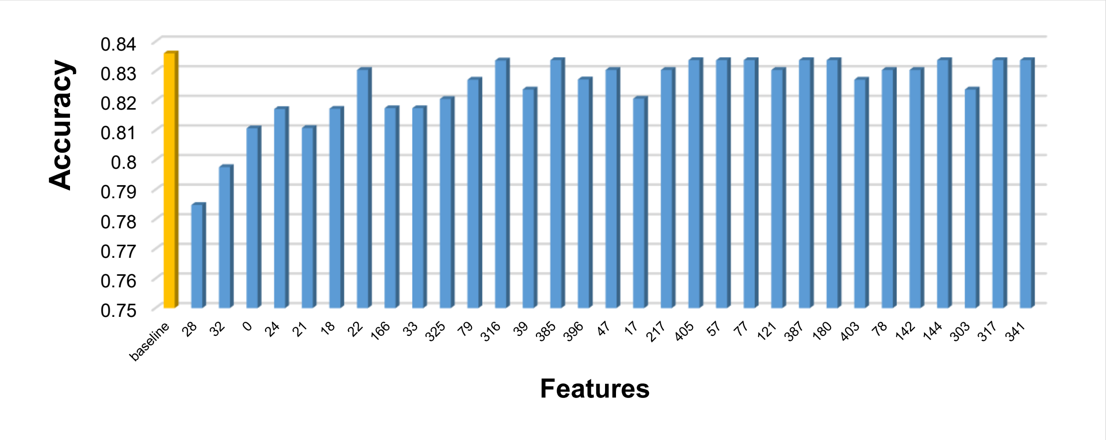

Supplementary Fig. 7. Cross-validation Accuracy (n=5 folds) in Feature Ablation Study for SVC. Baseline represents the scenario where no features have been deleted and is highlighted in yellow. Source data are provided as a Source Data file.

4. Details of the validation experiment

4.1 Sequences of validation experiment

The eight randomly generated cyclic peptides, cyclo-APYCGSI, cyclo-YLHIEGV, cyclo-KCL, cyclo-QSVQERS, cyclo-QEYQHEK, cyclo-HMHPW, cyclo-MMVMPY and cyclo-WMLWEPF were created using "Random Cyclopeptide Generator", our dedicated random sequence generator package. This tool employs a purely stochastic algorithm that: (1) randomly samples sequence lengths from 2 to 14 residues, and (2) independently selects each amino acid from the 20 native types with uniform probability distribution. The "Random Cyclopeptide Generator" package has been uploaded to our GitHub repository (https://github.com/Yongboxiao/Selection-of-Cyclization-Sites).

These randomly generated peptides, along with the selected *Pseudostellariae radix*-derived cyclic peptides, were processed using the "Cyclic Peptides Disconnection Sequences Generated" section of the program available in our GitHub repository to disconnect them at all cyclization sites, thereby generating the corresponding linear peptide sequences for synthesis. These sequences were subsequently input into the

“Prediction” section of the trained model for prediction. Experimental validation was performed to assess the accuracy of the model. The experimental process was consistent with the synthesis process in the dataset (see **Automated cyclic peptide synthesis**).

4.2 Details of sequence similarity evaluation

The training set comprised 70% of 306 cyclic peptide sequences, and no scaffold appeared in both training set and experimental set. For each test sequence, we computed the maximum global percent identity to any sequence in the training set by performing global pairwise alignments on the corresponding linearized peptides. Because cyclic peptides can be represented with different starting positions when linearized, we further conducted a rotation-invariant similarity audit for high-similarity pairs (initial identity ≥ 60%), in which all possible cyclic rotations of the peptide sequence were considered to ensure an unbiased assessment of sequence overlap. Percent identity was defined as the fraction of identical amino acids over the length of the longer sequence. Across the test set, the mean maximum percent identity was 34.4%, with 67.6% of test sequences showing <40% identity to any training sequence. The maximum observed identity was 75.0%, reflecting a small number of cross-scaffold peptides with high sequence resemblance due to the cyclic nature of the molecules.

Supplementary Table 5 Maximum percent identity between each experimental validation (test) sequence and the training set.

Experimental Sequences	Max Identity Training Sequences	Max Identity
MMVMPY	FEGMPN	33.33%
MVMPYM	EGMP	33.33%
VMPYMM	VMG	33.33%
MPYMMV	MPNS	33.33%
PYMMVM	PFGPTL	16.67%
YMMVMP	ITPVIPG	28.57%
WMLWEPF	NML	28.57%
MLWEPFW	QAMRPFW	42.86%
LWEPFWM	AMRPFW	42.86%
WEPFWML	MRPFW	42.86%

EPFWMLW	RPFW	42.86%
PFWMLWE	PFGPTL	28.57%
FWMLWEP	GPTLWPF	28.57%
QSVQERS	QPGQPG	28.57%
SVQERSQ	AFQYRCG	28.57%
VQERSQS	VQI	28.57%
QERSQSV	AYRGFAA	14.29%
ERSQSVQ	ERG	28.57%
RSQSVQE	RSM	28.57%
SQSVQER	SEAVDGTAK	22.22%
QEYQHEK	QPGQPG	28.57%
EYQHEKQ	DGTHYK	28.57%
YQHEKQE	GTHYK	28.57%
QHEKQEY	THYK	28.57%
HEKQEYQ	GAFQAY	28.57%
EKQEYQH	AFQAY	28.57%
KQEYQHE	KGDY	28.57%
HMHPW	AMRPFW	33.33%
MHPWH	MSPG	40.00%
HPWHM	HG	20.00%
PWHMH	GTHYK	20.00%
WHMHP	QAMRPFW	28.57%
GPVYA	GPSYFG	50.00%
PVYAG	TDDAG	40.00%
VYAGP	AYKG	40.00%
YAGPV	YQG	40.00%
AGPVY	IGPSYFG	42.86%
GGYPLIL	GITPVIP	42.86%
GYPLILG	ITPVIPG	42.86%
YPLILGG	TIDTLG	28.57%
PLILGGY	TFIL	28.57%
LILGGYP	LDPGTF	28.57%

ILGGYPL	ITPGIPG	42.86%
LGGYPLI	MGGGPNSFEG	30.00%
LAGPY	LDG	40.00%
AGPYL	FGPTL	60.00%
GPYLA	GPTL	75.00%
PYLAG	TDDAG	40.00%
YLAGP	WAAGG	40.00%
GTPTPLFF	GLPPPIFG	50.00%
TPTPLFFG	GPTLWPF	37.50%
PTPLFFGT	ITPVIPG	37.50%
TPLFFGTP	TDGFFG	50.00%
PLFFGTPT	ILDPGTF	37.50%
LFFGTPTP	LDPGTF	37.50%
FFGTPTPL	FEGMPN	37.50%
FGTPTPLF	PFGPTL	25.00%
APYCGSI	FAYRG	28.57%
PYCGSIA	AYRGFAA	42.86%
YCGSIAP	YRGFAA	42.86%
CGSIAPY	NGTKGDY	28.57%
GSIAPYC	FGIGPS	28.57%
SIAPYCG	AFQYRCG	28.57%
IAPYCGS	FAQYCRG	42.86%
LKC	HKV	33.33%
KCL	NML	33.33%
CLK	CLG	66.67%
GVYLHIE	GPTLWPF	28.57%
VYLHIEG	ITPVIPG	28.57%
YLHIEGV	YNSFPGM	28.57%
LHIEGVY	MSFEGMP	28.57%
HIEGVYL	WPFGPTL	28.57%
IEGVYLH	PFGPTL	28.57%
EGVYLHI	KGDY	28.57%

5. Introduction for the functions of “CycloPepper”

The **CycloPepper** is an intuitive tool designed to predict cyclization outcomes for head-to-tail cyclic peptides. It can be accessed both through software and a website, which is available for mobile use. On the website, users can input peptide sequences individually (Single mode), while the software supports both individual inputs and bulk uploads via a CSV file (Batch mode).

When in single mode **(Supplementary Fig. 8)**, upon clicking the **“GO”** button, the software rapidly generates predictions, classifying the overall result as **“SUCCESS”** (if at least one cyclization site is predicted to yield a cyclic product) or **“FAIL”** (if no viable sites are identified). The detailed results are displayed in table, where each row corresponds to a specific cyclization site. The **“Linear Seg.”** column lists all linearized peptide sequences generated by disconnecting the cyclic structure at each potential cyclization site. The **“Result”** column visually indicates the prediction for each site: a **green checkmark** denotes successful cyclization, while a **red cross** signifies failure. This dual-color coding ensures quick interpretation of outcomes, enabling researchers to prioritize synthesizable candidates efficiently.

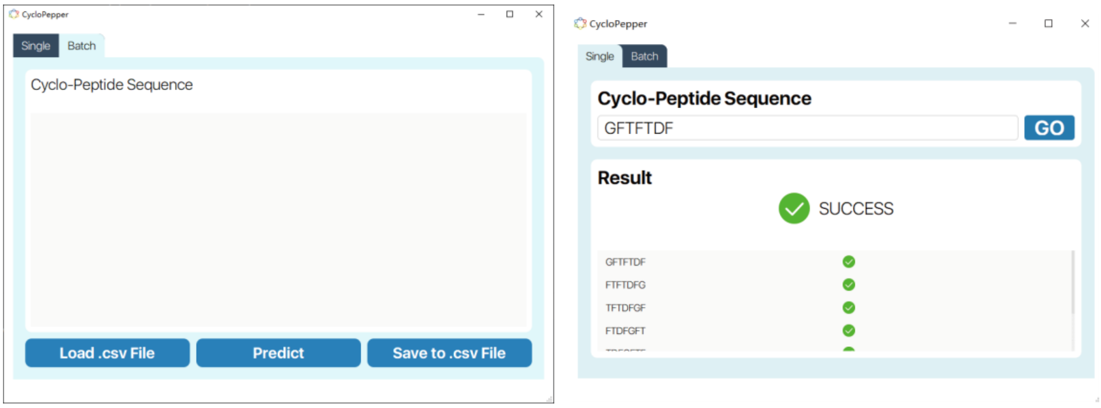

Supplementary Fig. 8. Interface of CycloPepper in single mode.

When in batch mode **(Supplementary Fig. 9)**, click the **“Load .csv File”** button, then select the CSV File that matches the format. Click **“Predict”** and all the sequences are processed in turn. The software rapidly generates predictions and detailed results are presented in table. The “**Cyclo**” column shows the cyclic peptide sequence, the **“linear”** column shows the linear peptide sequence corresponding to each cyclization

site, the **“result”** column shows the cyclization prediction result of each site. And the **“conclusion”** column shows the prediction result of the cyclic peptide sequence. If at least one site can produce a cyclic peptide product, it will display a **green checkmark.** Otherwise, it will display a **red cross**.

The usage of the CycloPepper website interface **(Supplementary Fig. 10)** is the same as the software’s single mode operation. The website is easily accessible on mobile phones, allowing predictions to be made anytime and anywhere, providing convenience for researchers.

The tool’s streamlined workflow and batch-processing capability make it ideal for high-throughput screening of therapeutic cyclic peptides, accelerating the design-validation cycle while minimizing experimental costs.

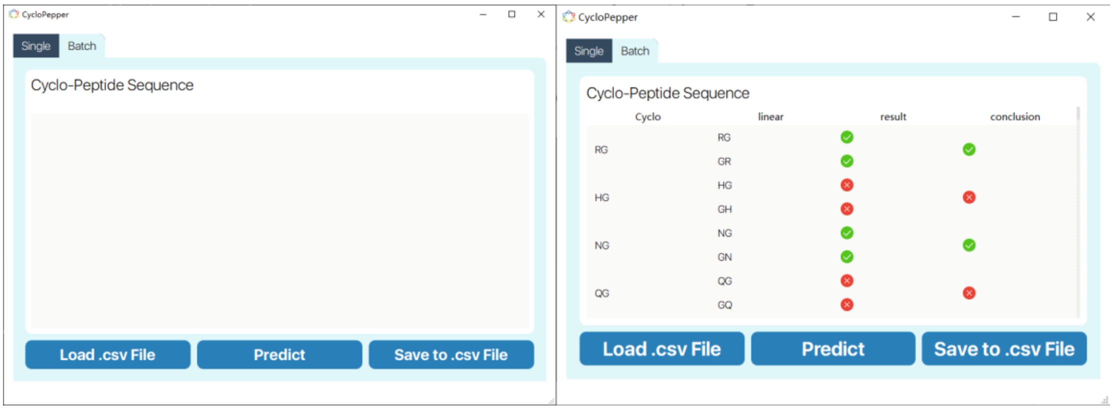

Supplementary Fig. 9. Interface of CycloPepper in batch mode.

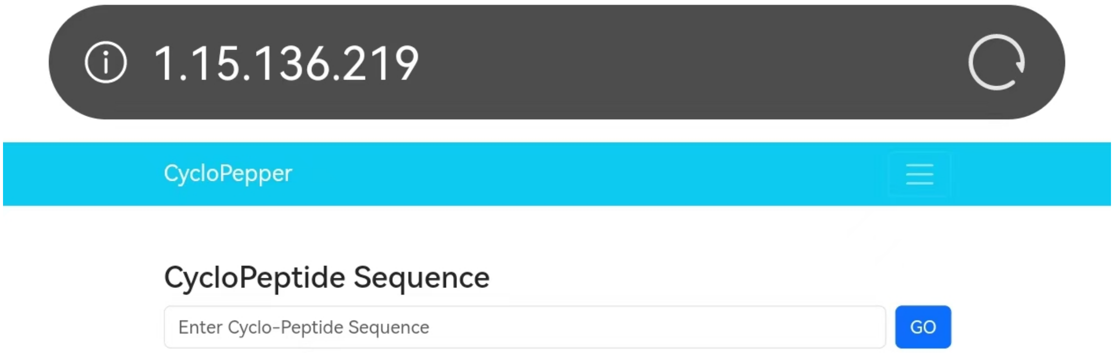

Supplementary Fig. 10. The CycloPepper website page accessed via mobile phone.

6. Procedures for predicting cyclization sites of cyclic peptides targeting various diseases

Antibodies corresponding to the targets previously mentioned were retrieved from the SAbDab database[3]. The sequences of their complementarity-determining regions (CDRs) were subsequently designed as cyclic peptides. Due to the presence of only six sequences with more than 13 residues in the dataset used for model training, CDR sequences exceeding 13 residues were excluded. In total, 561 cyclic peptide sequences and their corresponding scores are provided in **Supplementary Table 6**.

Supplementary Table 6. 561 cyclic peptide sequences targeting various diseases and scores.

Sequence	Score
Abciximab (ITGA2B/CD41)	
GYTFTNY	1
YTFTNYG	0
TFTNYGY	1
FTNYGYT	1
TNYGYTF	1
NYGYTFT	1
YGYTFTN	1
YPGNSD	1
PGNSDY	0
GNSDYP	1
NSDYPG	1
SDYPGN	1
DYPGNS	0
YDGYYVFAY	1
DGYYVFAYY	1
GYYVFAYYD	1

Sequence	Score
Anvatabart (ERBB2/CD340/HER2)	
GFNIKDT	1
FNIKDTG	1
NIKDTGF	1
IKDTGFN	1
KDTGFNI	1
DTGFNIK	0
TGFNIKD	1
YPTNGY	1
PTNGYY	0
TNGYYP	1
NGYYPT	1
GYYPTN	1
YYPTNG	0
WGGDGFYAMDY	1
GGDGFYAMDYW	1
GDGFYAMDYWG	1

Sequence	Score
YYVFAYYDG	1
YVFAYYDGY	1
VFAYYDGYY	1
FAYYDGYYV	1
AYYDGYYVF	1
YYDGYYVFA	1
RASRDISNNLH	1
ASRDISNNLHR	1
SRDISNNLHRA	1
RDISNNLHRAS	0
DISNNLHRASR	0
ISNNLHRASRD	1
SNNLHRASRDI	0
NNLHRASRDIS	1
NLHRASRDISN	1
LHRASRDISNN	1
HRASRDISNNL	0
YASQSMS	0
ASQSMSY	0
SQSMSYA	0
QSMSYAS	1
SMSYASQ	0
MSYASQS	0
SYASQSM	0
QQTNSWPYT	0
QTNSWPYTQ	0
TNSWPYTQQ	0
NSWPYTQQT	0
SWPYTQQTN	0
WPYTQQTNS	0
PYTQQTNSW	0

Sequence	Score
DGFYAMDYWGG	1
GFYAMDYWGGD	0
FYAMDYWGGDG	1
YAMDYWGGDGF	0
AMDYWGGDGFY	1
MDYWGGDGFYA	1
DYWGGDGFYAM	1
YWGGDGFYAMD	1
RASQDVNTAVA	0
ASQDVNTAVAR	0
SQDVNTAVARA	0
QDVNTAVARAS	0
DVNTAVARASQ	1
VNTAVARASQD	0
NTAVARASQDV	0
TAVARASQDVN	0
AVARASQDVNT	0
VARASQDVNTA	0
ARASQDVNTAV	0
SASFLYS	1
ASFLYSS	1
SFLYSSA	1
FLYSSAS	1
LYSSASF	1
YSSASFL	1
SSASFLY	1
QQHYTTPPT	0
QHYTTPPTQ	0
HYTTPPTQQ	0
YTTPPTQQH	0
TTPPTQQHY	0

Sequence	Score
YTQQTNSWP	0
TQQTNSWPY	0
Adalimumab (TNF/TNFA)	
GFTFDDY	1
FTFDDYG	1
TFDDYGF	1
FDDYGFT	1
DDYGFTF	1
DYGFTFD	1
YGFTFDD	1
TWNSGH	0
WNSGHT	0
NSGHTW	0
SGHTWN	1
GHTWNS	0
HTWNSG	0
VSYLSTASSLDY	1
SYLSTASSLDYV	1
YLSTASSLDYVS	1
LSTASSLDYVSY	1
STASSLDYVSYL	1
TASSLDYVSYLS	0
ASSLDYVSYLST	1
SSLDYVSYLSTA	0
SLDYVSYLSTAS	0
LDYVSYLSTASS	0
DYVSYLSTASSL	1
YVSYLSTASSLD	0
RASQGIRNYLA	1
ASQGIRNYLAR	1
SQGIRNYLARA	1

Sequence	Score
TPPTQQHYT	0
PPTQQHYTT	0
PTQQHYTTP	0
TQQHYTTPP	1
Pertuzumab (ERBB2/CD340/HER2)	
GFTFTDY	1
FTFTDYG	1
TFTDYGF	1
FTDYGFT	1
TDYGFTF	1
DYGFTFT	1
YGFTFTD	1
NPNSGG	1
PNSGGN	0
NSGGNP	0
SGGNPN	1
GGNPNS	0
GNPNSG	1
NLGPSFYFDY	1
LGPSFYFDYN	1
GPSFYFDYNL	1
PSFYFDYNLG	0
SFYFDYNLGP	1
FYFDYNLGPS	1
YFDYNLGPSF	0
FDYNLGPSFY	1
DYNLGPSFYF	1
YNLGPSFYFD	1
KASQDVSIGVA	1
ASQDVSIGVAK	1
SQDVSIGVAKA	1

Sequence	Score	Sequence	Score
QGIRNYLARAS	0	QDVSIGVAKAS	1
GIRNYLARASQ	1	DVSIGVAKASQ	1
IRNYLARASQG	1	VSIGVAKASQD	1
RNYLARASQGI	1	SIGVAKASQDV	1
NYLARASQGIR	1	IGVAKASQDVS	1
YLARASQGIRN	1	GVAKASQDVSI	1
LARASQGIRNY	0	VAKASQDVSIG	1
ARASQGIRNYL	0	AKASQDVSIGV	1
AASTLQS	1	SASYRYT	1
ASTLQSA	0	ASYRYTS	1
STLQSAA	0	SYRYTSA	1
TLQSAAS	0	YRYTSAS	1
LQSAAST	0	RYTSASY	1
QSAASTL	0	YTSASYR	1
SAASTLQ	0	TSASYRY	1
QRYNRAPYT	0	QQYYIYPYT	0
RYNRAPYTQ	0	QYYIYPYTQ	1
YNRAPYTQR	0	YYIYPYTQQ	0
NRAPYTQRY	1	YIYPYTQQY	1
RAPYTQRYN	0	IYPYTQQYY	0
APYTQRYNR	0	YPYTQQYYI	1
PYTQRYNRA	0	PYTQQYYIY	0
YTQRYNRAP	1	YTQQYYIYP	1
TQRYNRAPY	0	TQQYYIYPY	1
Infliximab (TNF/TNFA)		Arcitumomab (CEA/CEACAMs)	
GFIFSNH	0	GFTFTDY	1
FIFSNHG	0	FTFTDYG	1
IFSNHGF	1	TFTDYGF	1
FSNHGFI	0	FTDYGFT	1
SNHGFIF	0	TDYGFTF	1
NHGFIFS	0	DYGFTFT	1

Sequence	Score
HGFIFSN	0
RSKSINSA	1
SKSINSAR	0
KSINSARS	1
SINSARSK	1
INSARSKS	1
NSARSKSI	1
SARSKSIN	1
ARSKSINS	1
NYYGSTYDY	1
YYGSTYDYN	1
YGSTYDYNY	0
GSTYDYNYY	1
STYDYNYYG	1
TYDYNYYGS	0
YDYNYYGST	0
DYNYYGSTY	1
YNYYGSTYD	1
RASQFVGSSIH	1
ASQFVGSSIHR	1
SQFVGSSIHRA	1
QFVGSSIHRAS	0
FVGSSIHRASQ	1
VGSSIHRASQF	1
GSSIHRASQFV	1
SSIHRASQFVG	1
SIHRASQFVGS	1
IHRASQFVGSS	1
HRASQFVGSSI	1
YASESMS	1
ASESMSY	0

Sequence	Score
YGFTFTD	1
GNKANGYT	0
NKANGYTG	1
KANGYTGN	1
ANGYTGNK	1
NGYTGNKA	1
GYTGNKAN	0
YTGNKANG	1
TGNKANGY	1
DRGLRFYFDY	1
RGLRFYFDYD	1
GLRFYFDYDR	1
LRFYFDYDRG	0
RFYFDYDRGL	1
FYFDYDRGLR	1
YFDYDRGLRF	0
FDYDRGLRFY	1
DYDRGLRFYF	1
YDRGLRFYFD	1
RASSSVTYIH	1
ASSSVTYIHR	1
SSSVTYIHRA	0
SSVTYIHRAS	0
SVTYIHRASS	1
VTYIHRASSS	1
TYIHRASSSV	1
YIHRASSSVT	0
IHRASSSVTY	0
HRASSSVTYI	1
ATSNLAS	1
TSNLASA	1

Sequence	Score
SESMSYA	1
ESMSYAS	1
SMSYASE	1
MSYASES	1
SYASESM	1
QQSHSWPFT	0
QSHSWPFTQ	0
SHSWPFTQQ	0
HSWPFTQQS	0
SWPFTQQSH	0
WPFTQQSHS	0
PFTQQSHSW	0
FTQQSHSWP	0
TQQSHSWPF	0
Alemtuzumab (CD52)	
GFTFTDF	1
FTFTDFG	1
TFTDFGF	1
FTDFGFT	1
TDFGFTF	1
DFGFTFT	1
FGFTFTD	1
RDKAKGYT	1
DKAKGYTR	1
KAKGYTRD	1
AKGYTRDK	1
KGYTRDKA	1
GYTRDKAK	1
YTRDKAKG	1
TRDKAKGY	1
EGHTAAPFDY	1

Sequence	Score
SNLASAT	1
NLASATS	1
LASATSN	1
ASATSNL	1
SATSNLA	0
QHWSSKPPT	0
HWSSKPPTQ	0
WSSKPPTQH	0
SSKPPTQHW	0
SKPPTQHWS	0
KPPTQHWSS	1
PPTQHWSSK	0
PTQHWSSKP	0
TQHWSSKPP	0
Atezolizumab (PDL1/CD274)	
GFTFSDS	1
FTFSDSG	1
TFSDSGF	1
FSDSGFT	1
SDSGFTF	1
DSGFTFS	1
SGFTFSD	1
SPYGGS	1
PYGGSS	0
YGGSSP	1
GGSSPY	1
GSSPYG	1
SSPYGG	1
RHWPGGFDY	1
HWPGGFDYR	0
WPGGFDYRH	1

Sequence	Score
GHTAAPFDYE	1
HTAAPFDYEG	1
TAAPFDYEGH	1
AAPFDYEGHT	0
APFDYEGHTA	0
PFDYEGHTAA	0
FDYEGHTAAP	1
DYEGHTAAPF	1
YEGHTAAPFD	1
KASQNIDKYLN	0
ASQNIDKYLNK	1
SQNIDKYLNKA	1
QNIDKYLNKAS	1
NIDKYLNKASQ	1
IDKYLNKASQN	1
DKYLNKASQNI	0
KYLNKASQNID	1
YLNKASQNIDK	1
LNKASQNIDKY	1
NKASQNIDKYL	0
NTNNLQT	0
TNNLQTN	0
NNLQTNT	0
NLQTNTN	0
LQTNTNN	0
QTNTNNL	0
TNTNNLQ	0
LQHISRPRT	0
QHISRPRTL	1
HISRPRTLQ	1
ISRPRTLQH	1

Sequence	Score
PGGFDYRHW	0
GGFDYRHWP	1
GFDYRHWPG	1
FDYRHWPGG	0
DYRHWPGGF	1
YRHWPGGFD	1
RASQDVSTAVA	0
ASQDVSTAVAR	0
SQDVSTAVARA	0
QDVSTAVARAS	0
DVSTAVARASQ	1
VSTAVARASQD	0
STAVARASQDV	0
TAVARASQDVS	0
AVARASQDVST	0
VARASQDVSTA	0
ARASQDVSTAV	0
SASFLYS	1
ASFLYSS	1
SFLYSSA	1
FLYSSAS	1
LYSSASF	1
YSSASFL	1
SSASFLY	1
QQYLYHPAT	0
QYLYHPATQ	0
YLYHPATQQ	0
LYHPATQQY	0
YHPATQQYL	0
HPATQQYLY	1
PATQQYLYH	0

Sequence	Score
SRPRTLQHI	1
RPRTLQHIS	1
PRTLQHISR	0
RTLQHISRP	1
TLQHISRPR	0
Aducanumab (APP)	
GFAFSSY	1
FAFSSYG	1
AFSSYGF	1
FSSYGFA	1
SSYGFAF	1
SYGFAFS	1
YGFAFSS	1
WFDGTK	1
FDGTKW	0
DGTKWF	1
GTKWFD	1
TKWFDG	1
KWFDGT	1
RASQSISSYLN	1
ASQSISSYLNR	1
SQSISSYLNRA	1
QSISSYLNRAS	0
SISSYLNRASQ	1
ISSYLNRASQS	1
SSYLNRASQSI	0
SYLNRASQSIS	1
YLNRASQSISS	1
LNRASQSISSY	1
NRASQSISSYL	0
AASSLQS	0

Sequence	Score
ATQQYLYHP	1
TQQYLYHPA	0
Avelumab (PDL1/CD274)	
GFTFSSY	1
FTFSSYG	1
TFSSYGF	1
FSSYGFT	1
SSYGFTF	1
SYGFTFS	1
YGFTFSS	1
YPSGGI	1
PSGGIY	0
SGGIYP	1
GGIYPS	1
GIYPSG	1
IYPSGG	1
IKLGTVTTVDY	1
KLGTVTTVDYI	1
LGTVTTVDYIK	1
GTVTTVDYIKL	0
TVTTVDYIKLG	1
VTTVDYIKLGT	0
TTVDYIKLGTV	0
TVDYIKLGTVT	0
VDYIKLGTVTT	0
DYIKLGTVTTV	0
YIKLGTVTTVD	0
DVSNRPS	1
VSNRPSD	1
SNRPSDV	1
NRPSDVS	1

Sequence	Score
ASSLQSA	0
SSLQSAA	0
SLQSAAS	0
LQSAASS	0
QSAASSL	0
SAASSLQ	0
QQSYSTPLT	0
QSYSTPLTQ	0
SYSTPLTQQ	1
YSTPLTQQS	0
STPLTQQSY	0
TPLTQQSYS	1
PLTQQSYST	0
LTQQSYSTP	0
TQQSYSTPL	1
Ponezumab (APP)	
GYYTEAY	1
YYTEAYG	1
YTEAYGY	1
TEAYGYY	0
EAYGYYT	1
AYGYYTE	1
YGYYTEA	1
DPATGN	1
PATGND	1
ATGNDP	1
TGNDPA	1
GNDPAT	0
NDPATG	1
LYSLPVY	1
YSLPVYL	1

Sequence	Score
RPSDVSN	1
PSDVSNR	0
SDVSNRP	0
SSYTSSSTRV	0
SYTSSSTRVS	1
YTSSSTRVSS	1
TSSSTRVSSY	1
SSSTRVSSYT	0
SSTRVSSYTS	0
STRVSSYTSS	1
TRVSSYTSSS	1
RVSSYTSSST	0
VSSYTSSSTR	0
Itanistomig (PDL1/CD274)	
GDTFSTY	1
DTFSTYG	1
TFSTYGD	0
FSTYGDT	1
STYGDTF	1
TYGDTFS	1
YGDTFST	1
IPIFGK	0
PIFGKI	0
IFGKIP	0
FGKIPI	0
GKIPIF	0
KIPIFG	0
RASQSVSSYLA	1
ASQSVSSYLAR	1
SQSVSSYLARA	1
QSVSSYLARAS	0

Sequence	Score
SLPVYLY	1
LPVYLYS	0
PVYLYSL	0
VYLYSLP	1
YLYSLPV	0
QISRLDP	0
ISRLDPQ	0
SRLDPQI	0
RLDPQIS	1
LDPQISR	1
DPQISRL	1
PQISRLD	1
LQGTHYPVL	1
QGTHYPVLL	1
GTHYPVLLQ	0
THYPVLLQG	1
HYPVLLQGT	1
YPVLLQGTH	0
PVLLQGTHY	0
VLLQGTHYP	0
LLQGTHYPV	0

Sequence	Score
SVSSYLARASQ	1
VSSYLARASQS	1
SSYLARASQSV	1
SYLARASQSVS	1
YLARASQSVSS	1
LARASQSVSSY	1
ARASQSVSSYL	0
DASNRAT	1
ASNRATD	1
SNRATDA	1
NRATDAS	1
RATDASN	1
ATDASNR	1
TDASNRA	0
QQRSNWPT	0
QRSNWPTQ	0
RSNWPTQQ	0
SNWPTQQR	0
NWPTQQRS	0
WPTQQRSN	0
PTQQRSNW	0
TQQRSNWP	0

7. References

[1] F. Wan, C. Hu, P. Xie, X. Yang, Y. Pan, X. He, Z. Ning, C. Li, *J. Am. Chem. Soc.* **2025**, https://doi.org/10.1021/jacs.5c16902.

[2] B. H. Gless, P. Peng, K. D. Pedersen, C. H. Gotfredsen, H. Ingmer, C. A. Olsen, *Org. Lett.* **2017**, *19*, 5276.

[3] J. Dunbar, K. Krawczyk, J. Leem, T. Baker, A. Fuchs, G. Georges, J. Shi, C. M. Deane, *Nucleic Acids Res.* **2014**, *42*, D1140.

8. Synthetic LC-MS data for validation experiments

The following are the crude total ion current (TIC) chromatograms for each cyclic peptide, with irrelevant background peaks, such as the DIEA signal (0-2 minutes), excluded.

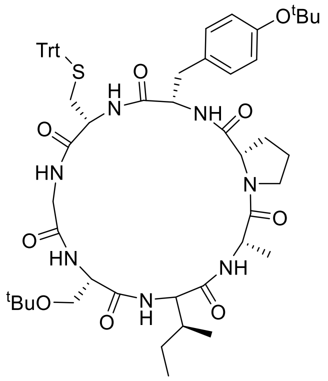

(a) Cyclo-APYCGSI

LC-MS condition: Method A. MS (ESI) exact mass calcd. for $C_{58}H_{75}N_7O_9S$, $[M+H]^+$: 1046.54; $[M+Na]^+$: 1068.52. Found: $[M+H]^+$: 1046.44; $[M+Na]^+$: 1068.42.

Entry	Sequence	Experimental score	Predicted score
1	APYCGSI	1	1
2	PYCGSIA	0	0
3	YCGSIAP	1	1
4	CGSIAPY	1	0
5	GSIAPYC	1	1
6	SIAPYCG	1	1
7	IAPYCGS	1	1

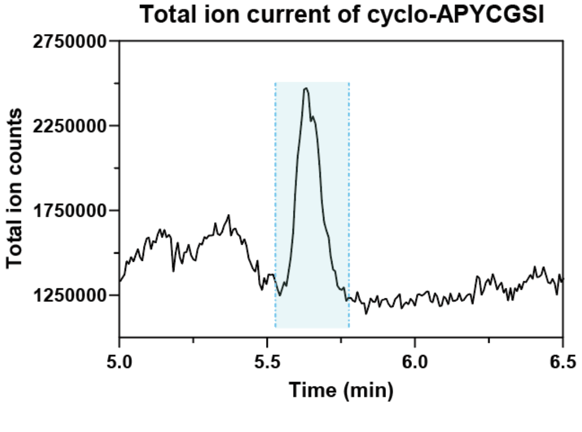

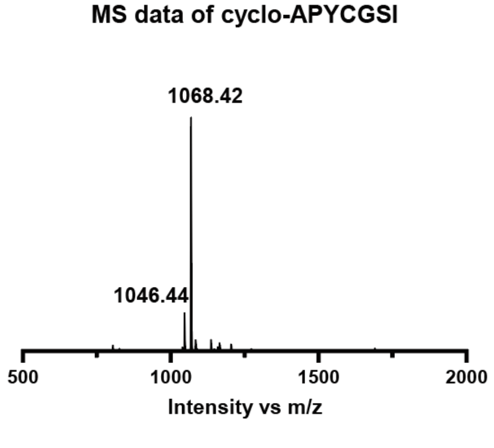

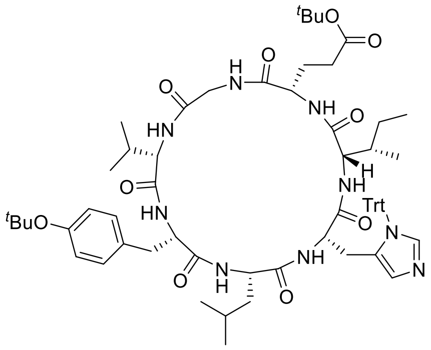

(b) Cyclo-YLHIEGV

LC-MS condition: Method B. MS (ESI) exact mass calcd. for $C_{66}H_{87}N_9O_{10}$, $[M+H]^+$: 1166.67; $[M+Na]^+$: 1188.65. Found: $[M+H]^+$: 1166.60.

Entry	Sequence	Experimental score	Predicted score
1	YLHIEGV	1	1
2	LHIEGVY	1	1
3	HIEGVYL	1	1
4	IEGVYLH	1	1
5	EGVYLHI	1	1
6	GVYLHIE	1	0
7	VYLHIEG	1	1

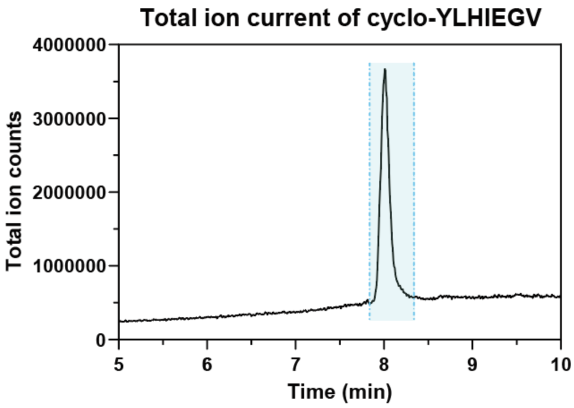

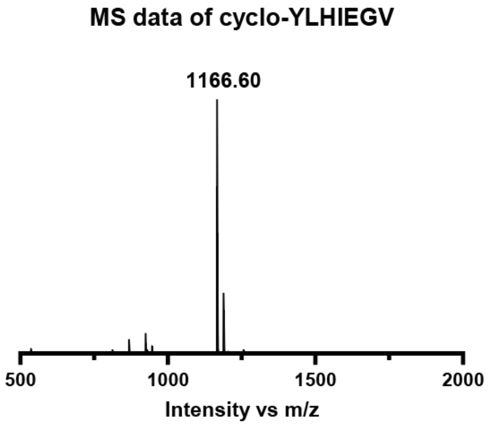

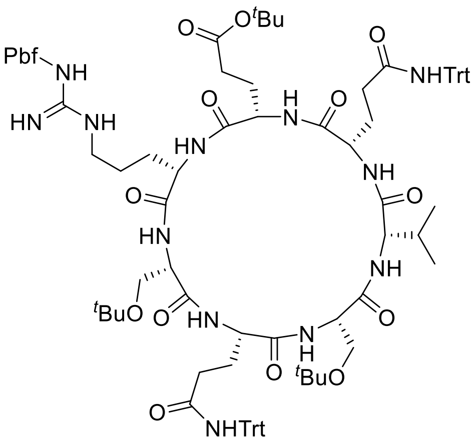

(d) Cyclo-QSVQERS

LC-MS condition: Method B. MS (ESI) exact mass calcd. for $C_{95}H_{122}N_{12}O_{16}S$, $[M+H]^+$: 1719.8822. Found: $[M+Na]^+$: 1741.98; $[(M+H+Na)/2]^+$: 871.61.

Entry	Sequence	Experimental score	Predicted score
1	QSVQERS	0	0
2	SVQERSQ	0	0
3	VQERSQS	0	0
4	QERSQSV	0	0
5	ERSQSVQ	0	0
6	RSQSVQE	1	1
7	SQSVQER	0	0

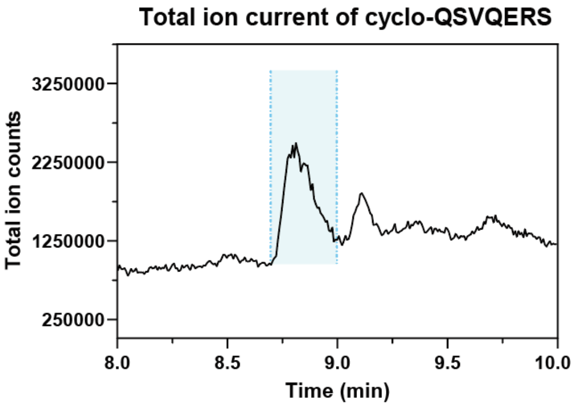

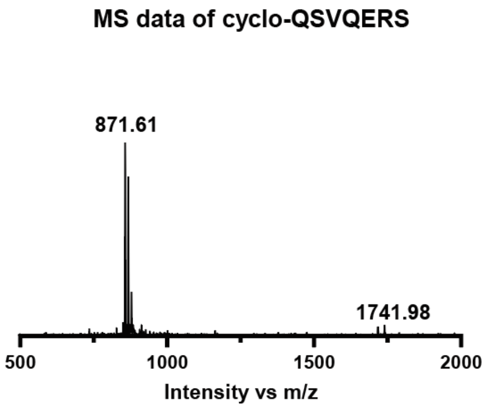

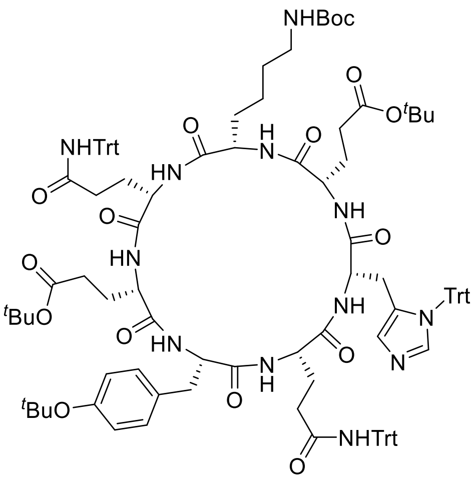

(e) Cyclo-QEYQHEK

LC-MS condition: Method B. MS (ESI) exact mass calcd. for $C_{115}H_{132}N_{12}O_{16}$, $[M+H]^+$: 1937.9884. Found: $[(M+2H)/2]^+$: 969.26; $[(M+H+Na)/2]^+$: 981.98; $[(M+2Na)/2]^+$: 992.39.

Entry	Sequence	Experimental score	Predicted score
1	QEYQHEK	0	0
2	EYQHEKQ	0	0
3	YQHEKQE	0	0
4	QHEKQEY	0	0
5	HEKQEYQ	1	1
6	EKQEYQH	1	1
7	KQEYQHE	0	0

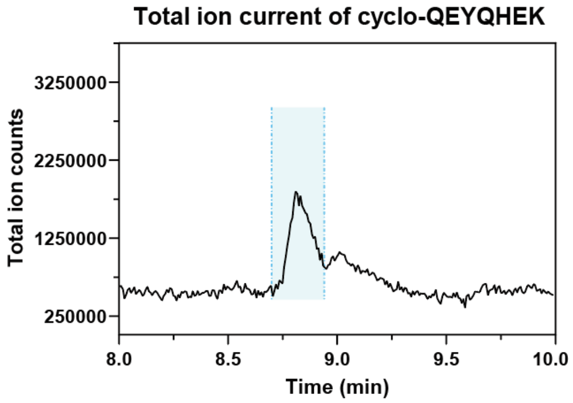

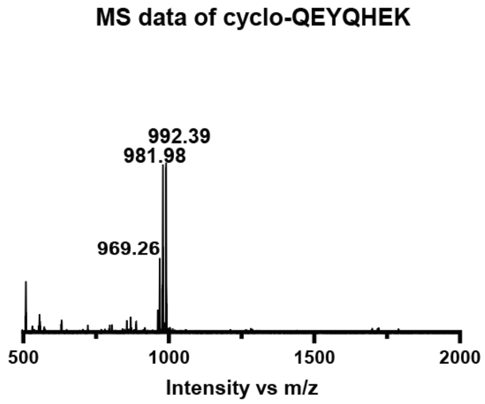

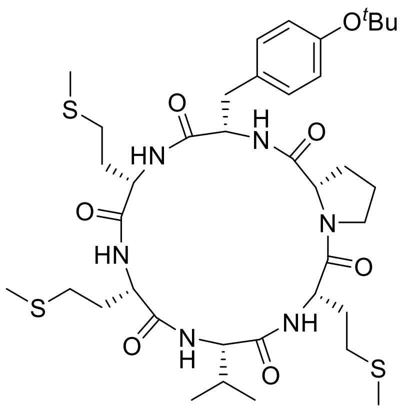

(g) Cyclo-MMVMPY

LC-MS condition: Method B. MS (ESI) exact mass calcd. for $C_{38}H_{60}N_6O_7S_3$, $[M+H]^+$: 809.3686. Found: $[M+H]^+$: 809.35; $[M+Na]^+$: 831.41.

Entry	Sequence	Experimental score	Predicted score
1	MMVMPY	1	1
2	MVMPYM	1	1
3	VMPYMM	0	0
4	MPYMMV	0	0
5	PYMMVM	0	0
6	YMMVMP	0	0

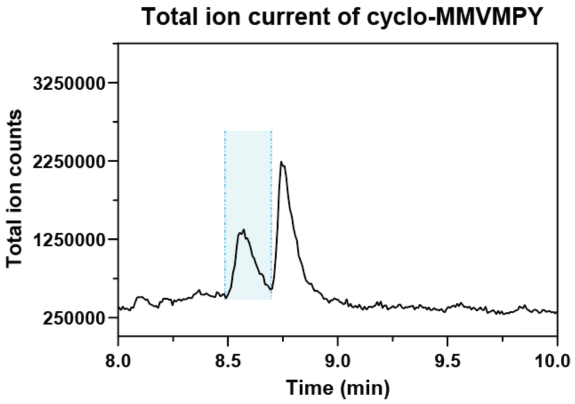

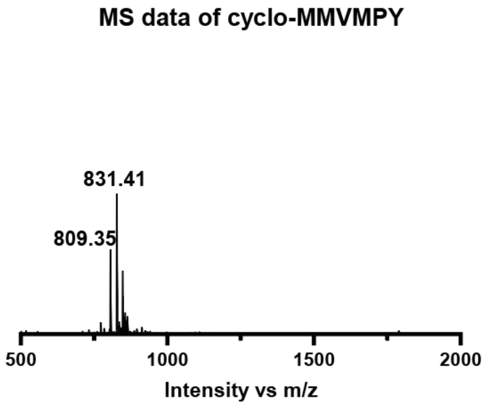

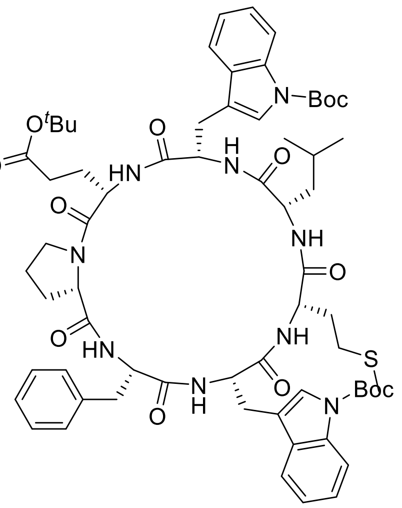

(h) Cyclo-WMLWEPF

LC-MS condition: Method B. MS (ESI) exact mass calcd. for $C_{66}H_{87}N_9O_{13}S$, $[M+H]^+$: 1246.6144. Found: $[M+H]^+$: 1246.69; $[M+Na]^+$: 1268.72.

Entry	Sequence	Experimental score	Predicted score
1	WMLWEPF	0	0
2	MLWEPFW	1	1
3	LWEPFWM	0	0
4	WEPFWML	1	1
5	EPFWMLW	0	0
6	PFWMLWE	0	0
7	FWMLWEP	1	0

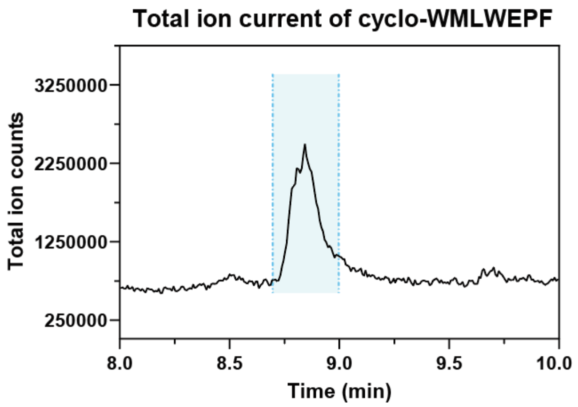

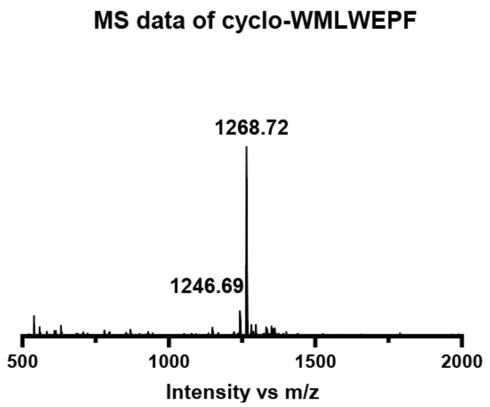

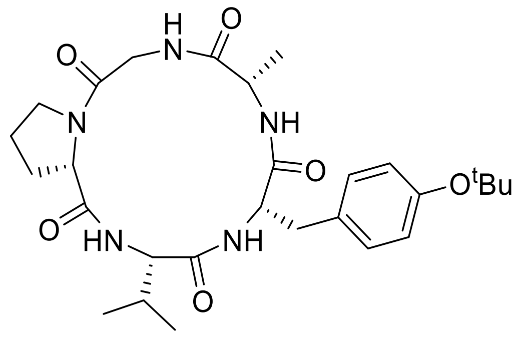

(i) Cyclo-GPVYA

LC-MS condition: Method A. MS (ESI) exact mass calcd. for $C_{28}H_{41}N_5O_6$, $[M+H]^+$: 544.31; $[M+Na]^+$: 566.30. Found: $[M+H]^+$: 544.37; $[M+Na]^+$: 566.31; $[2M+Na]^+$: 1109.50.

Entry	Sequence	Experimental score	Predicted score
1	GPVYA	1	1
2	PVYAG	0	0
3	VYAGP	0	0
4	YAGPV	0	0
5	AGPVY	0	0

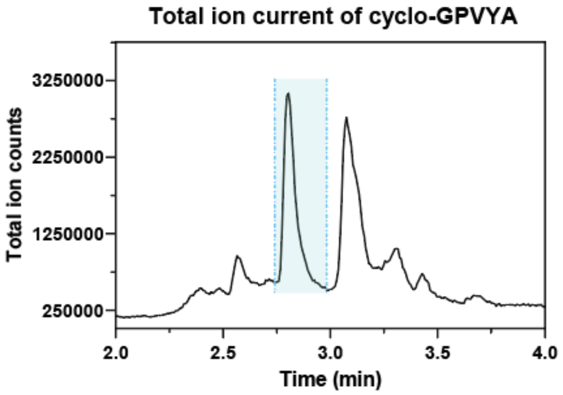

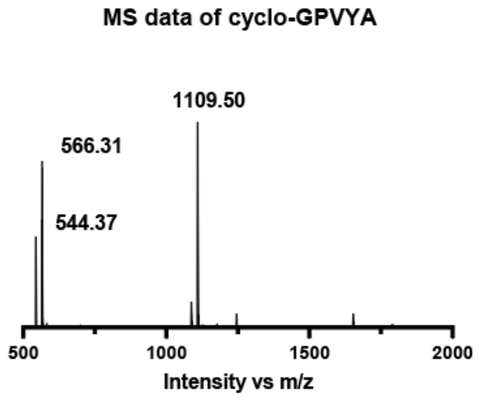

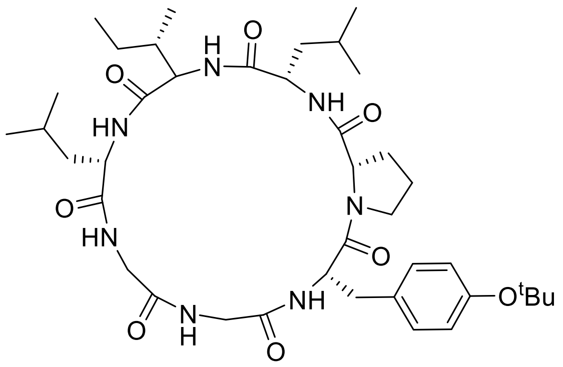

(j) Cyclo-GGYPLIL

LC-MS condition: Method A. MS (ESI) exact mass calcd. for $C_{40}H_{63}N_7O_8$, $[M+H]^+$: 770.48; $[M+Na]^+$: 792.46. Found: $[M+H]^+$: 770.43; $[M+Na]^+$: 792.42; $[2M+Na]^+$: 1562.80.

Entry	Sequence	Experimental score	Predicted score
1	GGYPLIL	1	1
2	GYPLILG	1	1
3	YPLILGG	1	1
4	PLILGGY	0	0
5	LILGGYP	1	1
6	ILGGYPL	1	1
7	LGGYPLI	0	1

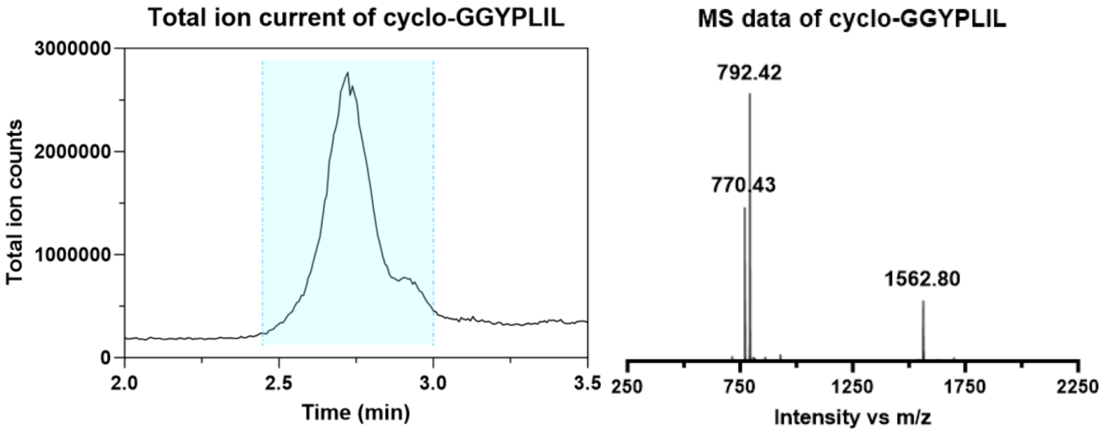

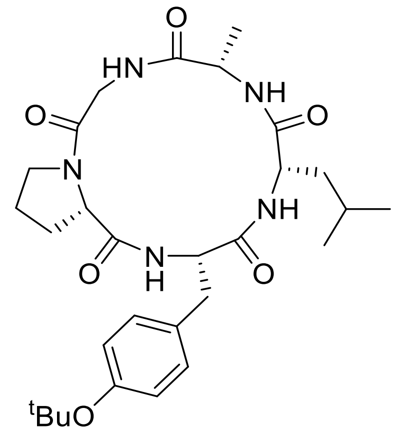

(k) Cyclo-LAGPY

LC-MS condition: Method A. MS (ESI) exact mass calcd. for $C_{29}H_{43}N_5O_6$, $[M+H]^+$: 558.33; $[M+Na]^+$: 580.31. Found: $[M+H]^+$: 558.37; $[2M+Na]^+$: 1137.44.

Entry	Sequence	Experimental score	Predicted score
1	LAGPY	1	1
2	AGPYL	1	1
3	GPYLA	1	1
4	PYLAG	1	0
5	YLAGP	1	1

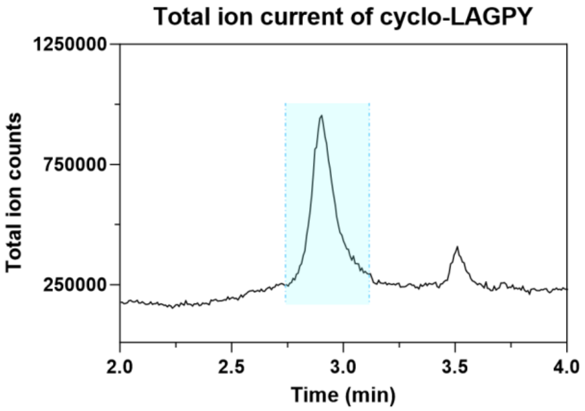

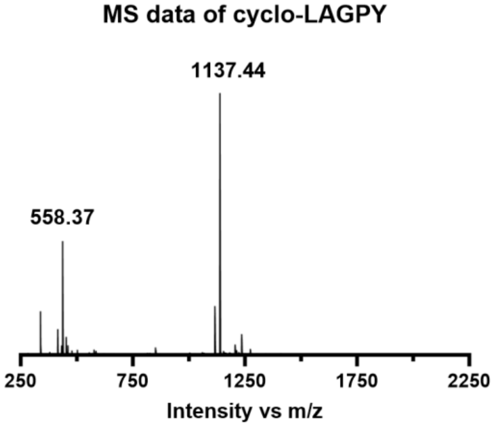

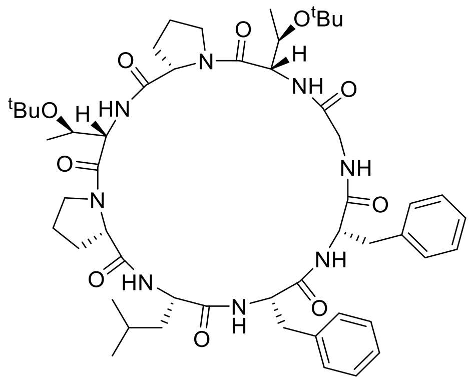

(l) Cyclo-GTPTPLFF

LC-MS condition: Method A. MS (ESI) exact mass calcd. for $C_{52}H_{76}N_8O_{10}$, $[M+H]^+$: 973.58; $[M+Na]^+$: 995.56. Found: $[M+Na]^+$: 995.47.

Entry	Sequence	Experimental score	Predicted score
1	GTPTPLFF	1	1
2	TPTPLFFG	1	1
3	PTPLFFGT	1	0
4	TPLFFGTP	1	1
5	PLFFGTPT	1	0
6	LFFGTPTP	1	1
7	FFGTPTPL	1	1
8	FGTPTPLF	1	1

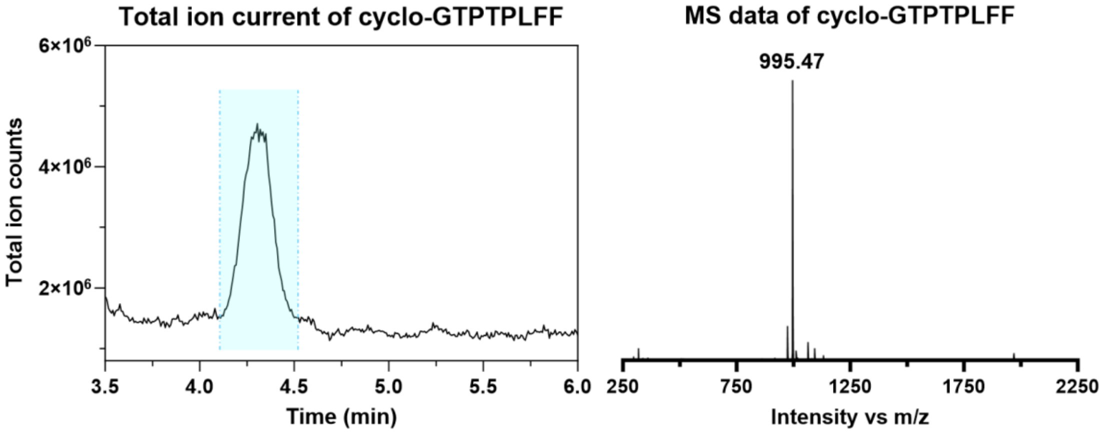

Supplement: Supplementary file 1 — Supplementary Information [file 41467_2026_69441_MOESM1_ESM.pdf]
